# A Universal Mental Health–Promoting Mobile App for Adolescents: Protocol for a Cluster Randomized Controlled Trial

**DOI:** 10.2196/42119

**Published:** 2023-01-11

**Authors:** Sabine Kaiser, Marte Rye, Reidar Jakobsen, Monica Martinussen, Helene Høgsdal, Henriette Kyrrestad

**Affiliations:** 1 Regional Centre for Child and Youth Mental Health and Child Welfare - North Faculty of Health Sciences UiT The Arctic University of Norway Tromsø Norway; 2 Department of Clinical Psychology Faculty of Psychology University of Bergen Bergen Norway

**Keywords:** mental health promotion, mobile app, mobile phone, adolescents, Opp, teens, application, effectiveness, intervention, effect, health, health promotion

## Abstract

**Background:**

In times of increasing mental health problems among young people, strengthening efforts to improve mental health through mental health promotion and prevention becomes increasingly important. Effective measures that support young people in coping with negative thoughts, feelings, and stress are essential, not just for the individual but also for society.

**Objective:**

The aim of this paper is to provide a description of a cluster randomized controlled trial that will be conducted to examine the effectiveness of Opp, a universal mental health–promoting mobile app for adolescents aged 13 to 19 years that provides information and exercises to better cope with stress, negative thoughts, and negative feelings. The protocol was developed in accordance with the SPIRIT checklist.

**Methods:**

An effectiveness study will be conducted with 3 measurement points: preintervention (T1), 2 weeks after the intervention (T2), and about 1 month after the intervention (T3). Adolescents will be recruited from middle and high schools in Norway and randomly assigned to the intervention or control groups. Randomization will be conducted on the school level. Opp can be downloaded from the Google Play or App Store but is password protected with a 4-digit code, which will be removed after study completion. Participants in the intervention group will receive a text message with the code to unlock the app. The participants in the intervention group can use Opp without limits on length or time of use. Objective data on how long or how often the participants use the app will not be collected. However, the second and third questionnaires for the intervention group contain app-specific questions on, for example, the use of the app.

**Results:**

Recruitment and data collection started in August and September 2022. So far, 381 adolescents have answered the first questionnaire. Data collection was expected to end in December 2022 but has had to be prolonged to approximately June 2023. The results of the study will be available in 2023 at the earliest.

**Conclusions:**

This project will contribute unique knowledge to the field, as there are few studies that have examined the effects of universal health-promoting mobile apps for adolescents. However, several limitations have to be taken into account when interpreting the results, such as randomization on the school level, the short time frame in which the study was conducted, and the lack of objective data to monitor the use of the app.

**Trial Registration:**

ClinicalTrials.gov NCT05211713; https://www.clinicaltrials.gov/ct2/show/NCT05211713

**International Registered Report Identifier (IRRID):**

PRR1-10.2196/42119

## Introduction

### Background

The World Health Organization defines mental health as a “state of well-being in which the individual realizes his or her own abilities, can cope with the normal stresses of life, can work productively and fruitfully, and is able to make a contribution to his or her community” [1]. Good mental health is essential to good health in general and is strongly related to quality of life and participation in school and leisure activities. A scoping review that underlined how difficult it is to define and operationalize good mental health identified 14 core domains of good mental health in young people, including attitudes toward mental disorders, self-perceptions, emotions, behaviors, self-management strategies, social skills, family and significant relationships, and quality of life [2]. The authors wrote that mental health promotion “leads to increasing well-being, competence and resilience and makes individuals improve their mental health and increase their control over it” [2]. Mental health promotion focuses on promoting the positive factors associated with mental health. On the individual level, these factors are, for example, coping with negative thoughts, negative feelings, and stress, as well as increasing resilience and self-esteem [3]. The World Health Organization defines health promotion as “the process of enabling people to increase control over, and to improve their health” [4].

It is widely acknowledged that public health work should focus on universal health-promoting and preventive measures rather than on the treatment of disorders [5,6]. Preventing mental disorders and promoting good mental health is more cost-effective for society and has greater benefits for public health [7]. Working preventively and promoting good mental health is particularly important for young people. On the one hand, adolescence is a vulnerable period in which young people transition from being children to being adults; many mental disorders develop during this period [8]. On the other hand, adolescence is a time when young people can learn and adopt healthy habits and choices from which they can benefit throughout their lives.

In order to reach adolescents, mental health–promoting interventions should be conducted where adolescents actually spend their time. This can, for example, be at schools, at places where adolescents carry out their leisure activities, or online. In Norway, most adolescents have their own smartphones, which they use daily and often for multiple hours [9]. This may open new opportunities to implement mental health–promoting interventions through mobile apps. Despite potential barriers, apps can reach a large proportion of adolescents. This is especially important in countries like Norway that are geographically large and where health services are not always easily accessible. Furthermore, apps are also cost-effective and might be appealing to adolescents, who have a relatively high threshold for seeking help face-to-face [10]. Adolescents prefer to be anonymous, and app-based interventions may therefore be important for promoting good mental health [11]. Also, adolescents often look for health-related information online [9]. However, the quality of the information they find can vary. It is therefore important to offer information that is both high quality and easily accessible to adolescents, especially when it comes to mental health.

Reviews from 2013 and 2014 [12,13] concluded that digital self-management resources were promising, but that there was a need for more research. A recently conducted meta-review that synthesized the results of 14 meta-analyses concluded, on the other hand, that mobile phone–based interventions already have potential for mental health promotion, especially when it comes to reducing psychological symptoms (ie, stress, anxiety, and depression) and increasing quality of life [14]. Although the effects of apps are generally of small magnitude, they could have an important societal impact if they were made available to large groups.

Kenny et al [15] conducted the first and, to the best of our knowledge, only cluster randomized controlled trial to examine the effectiveness of a mental health mobile app–based intervention in a general population of adolescents. They recruited 560 subjects aged 15 to 18 years from 10 schools that were randomly assigned to an intervention or control condition. Participants in the intervention group used the app for about 4 weeks. However, no significant differences in well-being, emotional distress, emotional self-awareness, or coping strategies were found.

One of the largest cluster randomized controlled trials of the effectiveness of universal school-based mindfulness training on mental health and well-being in early adolescence, the MYRIAD (My Resilience in Adolescence) project, found no evidence that it was more effective than the provision of usual social-emotional learning [16]. A meta-analysis of the effectiveness of relaxation techniques to reduce distress, anxiety, and depression in adolescents, on the other hand, had promising results with small to moderate effect sizes [17].

Identifying effective interventions for improving mental health among adolescents is necessary, and studies that have no or negative results also play an important role in determining what helps to improve mental health and what does not [18]. Or as Kuyken et al [16] conclude, “There is need to ask what works, for whom and how, as well as considering key contextual and implementation factors.”

### The Current Paper

The aim of the current paper is to describe a cluster randomized controlled trial that will be conducted to evaluate the effectiveness of Opp. Opp is a universal mental health–promoting mobile app for adolescents that provides information about mental health, feelings, and help seeking, as well as exercises and techniques to better cope with negative thoughts, negative feelings, and stress.

Opp is part of the research project UngRisk, which aims to develop and evaluate 2 mental health–promoting mobile apps that will be made available as free resources for easy access by young people. The other mobile app is called NettOpp and is aimed at young people aged 11 to 16 years who have experienced negative incidents online. A study similar to the one that is described here has already been conducted to examine NettOpp’s effectiveness [19]. Data analysis is in progress.

## Methods

### Eligibility Criteria and Setting of the Effectiveness Study

Both the adolescents and participating schools are convenience samples. Adolescents aged 13 to 19 years are eligible for participation in the study and will be recruited through middle and high schools in Norway.

### Inclusion and Exclusion Criteria

Adolescents aged 13 to 15 years need consent from their guardians, while adolescents that are 16 years or older can consent for themselves. Adolescents need a smartphone to use the app and need to be able to read Norwegian.

### Intervention

Opp is a universal mental health–promoting mobile app that is aimed at adolescents aged 13 to 19 years. The Norwegian word *opp* means “up” in English. In Norwegian, *opp* also evokes the word *opplysning*, which means “enlightenment” or “information”; *opp* also evokes the word “uplifting,” meaning to lift someone’s spirit.

The overall goals of Opp are to (1) increase well-being and mental health, (2) increase knowledge about mental health, (3) increase coping skills to deal with stress, (4) increase help-seeking behavior, (5) increase self-esteem, (6) increase sleep quality, and (7) reduce mental health problems.

To achieve these goals, Opp is theoretically divided into psychoeducational and resource modules (modules 1 and 2, respectively). Module 1 contains information about mental health (eg, “What is mental health?” “How are thoughts, feelings, and behavior connected?” “Why is good mental health important?” “How to get and maintain good mental health?” “What are mental health problems?” and “When should one seek help for their challenges?”), about feelings (eg, “What are feelings?” “Why are feelings important?” “Can you control your feelings?” and “What do you do when feelings take over?”), and about help seeking (eg, “When should you seek help?” “How to tell somebody about problems?” “What should happen when you have told somebody about your problems?” and “Here you can get help”).

Module 2 provides exercises and techniques on how to cope with difficult thoughts, feelings, and stress. For the latter, exercises include breathing and meditation exercises with animation and sound. Another exercise is inspired by cognitive behavioral therapy and aims at helping adolescents to recognize and challenge negative thoughts. Adolescents are guided through this exercise and can choose different statements (eg, “Nobody likes me”), how this thought makes them feel (eg, “sad”), and alternative thoughts (eg, “There are people who love and care about me”). In addition, there is an exercise in which the adolescents can choose different situations, choose what they might think and feel if they were in such situations, and choose how they would behave. This exercise also aims at raising awareness about the relationship between thoughts, feelings, and behavior. In addition, the app contains sleep hygiene advice, tips for maintaining good social relationships, and suggestions on how to increase self-esteem. The information is displayed through text, sound, and animation.

Opp can be downloaded from the Google Play and App Store, but the app is password protected. The password will be removed after study completion to make the app freely available to all users. Adolescents in the intervention group that have answered the first questionnaire can install Opp on their phone and will be provided with a 4-digit code via text message to unlock the app. It is up to the young people themselves to decide when they want to use the app and how much time they want to spend using it. It is not possible to monitor how much time the adolescents actually spend using Opp. However, there are app-specific questions in the second and third questionnaires, which the adolescents will be asked to fill in after approximately 2 and 4 weeks, respectively, that ask how often the adolescents have used the app. The participants are not provided with any training in using the app, but are instructed via text message on how to download the app. Also, the participants are given a phone number that they can call or send a text message to if they experience any problems with downloading or using the app.

### User Involvement

Users, that is, adolescents aged 13 to 19 years, were involved in the project from the development of the content of the intervention to its evaluation. Approximately 15 adolescents were involved in 2 workshops to provide input on the content, design, and name of the app. Two adolescents critically read through a manuscript that described the content of Opp and provided feedback on wording and terminology. There was also an extended reference group consisting of teachers, school-health nurses, nurses from a health care center for adolescents, and a community psychologist who contributed to the development of Opp through a workshop.

In addition to this, 27 adolescents participated in a user survey. The study indicated that Opp had good usability, and the overall mean star ranking of the app was 3.78 (SD 0.42) on a 5-point scale. A large proportion of the adolescents thought that Opp was interesting to use (20/27, 74%), that the design was good (25/27, 93%), and that it was appropriate for the target group (24/27, 89%) [20].

### Control

The waiting-list control group will receive the code to unlock the app after study completion.

### Randomization

This study is a cluster randomized controlled trial with intervention and waiting-list control groups. Schools will be randomly assigned to one of the groups by a statistician after baseline assessment. SPSS (IBM Corp) will be used to generate a random number between 0 and 1 that will be assigned to each school. The half of the schools with lower values for the random number will be assigned to the control group and the half of the schools with higher values will be assigned to the intervention group.

### Blinding

Neither schools, guardians, nor adolescent participants will know if they are assigned to the intervention or the control group prior to the baseline assessment.

### Outcomes

Data will be collected at 3 measurement points: baseline (T1; preintervention); approximately 2 weeks after the intervention (T2; postintervention); and approximately 1 month after the intervention (T3; follow-up) through self-report measures that the adolescents fill in using the secure online tool Nettskjema (University of Oslo).

### Primary Outcomes

Mental health will be assessed using the World Health Organization–5 Well-being Index (WHO-5) [21] and the Strengths and Difficulties Questionnaire (SDQ) [22]. Both the WHO-5 [23] and the SDQ [24] have been found to have adequate validity as outcome measures. The primary hypothesis is that there will be significant differences in changes in the mental health scores of the intervention and the control groups in favor of the intervention group.

### Secondary Outcomes

Coping skills will be measured with the Norwegian version of the General Self-Efficacy Scale [25]. A Norwegian study found that this scale had satisfactory internal consistency, test-retest reliability, and factor structure [26]. Help-seeking behavior will be assessed with 3 items that measure if the adolescents sought help, who they sought help from, and if they received the help they needed. The Self-liking and Competence Scale [27,28] will be used to measure self-liking and self-competence; this scale has been found to have good internal consistency and factor structure in a Norwegian validation study [29]. Sleep quality will be measured with six questions from the Bergen Child Study [30].

In addition to these measures, there will be app-specific questions in the second and third questionnaires for the intervention group that ask about how often and for how long the adolescents used the app and what features of the app they liked best.

### Power Calculations

Power calculations were conducted with PASS 16 (NCSS, LLC). Using multilevel analysis with a significance level of .05 will require a total sample of 1250 adolescents (625 in both the intervention and control groups, including 25 schools with 25 students per school) to achieve a power of .79 and to detect a small effect corresponding to Cohen *d*=0.20 when the expected interclass correlation at the school level is 0.02. Anticipating a drop-out rate of approximately 25%, a total of 1563 adolescents should be recruited [15].

### Data Management

Data will be collected with the secure Nettskjema platform and will be directly sent to and stored on a secure server that only members of the research team have access to. Data collection, data cleaning, and statistical analyses will also only be performed by members of the research team. The statistical analyses will be conducted on anonymized data from the secure server.

### Planned Statistical Analysis

Data analyses will be conducted using SPSS. Attrition analyses will be conducted to examine differences between those who complete the questionnaires and those who do not at different time points. A missing-values analysis will also be conducted. Variables for which there are a significantly greater number of missing values will be included as covariates in the analyses.

Correlations will be used to examine whether the different outcomes are related to each other at T1, and linear mixed models will be used to test the hypotheses. We will run 3-level mixed models with time nested within participants nested within schools, and model repeated measures using the *repeated* command in SPSS. We will assess the covariance structure of the repeated measurements by comparing models with different structures (ie, unstructured, compound symmetric, first-order auto-regressive, or others) using the Akaike information criterion.

### Ethics Approval and Consent to Participate

The study, including the information letter sent to the guardians and study participants and the consent forms, has been approved by the regional Research Ethics Committee (279207) and by the Norwegian Center for Research Data (715456). The study has been registered on ClinicalTrials.gov (NCT05211713). Active parental consent from 2 guardians is required for adolescents that are younger than 16 years to participate in the effectiveness study. Adolescents that are aged 16 years or older can consent by themselves. Consent, as well as the answers adolescents provide, will be collected digitally using Nettskjema and stored using the services for sensitive data of the University of Oslo in Norway [31]. It is possible that there will be unintended adverse effects of using the app or answering the survey that adolescents might find distressing [18]. Therefore, we will provide information about where the adolescents can receive help if they need it in the app, in the information letter, and at the end of the survey. Participants that fill in the 3 questionnaires will be entered in a raffle for 1 of 100 gift cards with a value of NOK 300 (approximately US $29). The app itself does not collect personal or sensitive information about the users, nor does it need access to GPS, the camera, contact lists, or anything else on the mobile phone.

## Results

Recruitment and data collection started in August and September 2022. However, teachers were on strike at that point for about 6 weeks, which made recruitment challenging as the schools were partly closed. Data collection was expected to end in December 2022 but had to be prolonged until approximately June 2023. So far, 381 adolescents have answered the first questionnaire. The results of the study will be available at the earliest at the end of 2023 or later, and will be published in international and national journals and presented at conferences.

## Discussion

### Principal Aims

The current paper describes a cluster randomized controlled trial that is being conducted to evaluate the effectiveness of a universal mental health–promoting mobile app (Opp) in Norway. Its evaluation will lead to new knowledge in the field, as there have been only a few studies published on the effectiveness of such universal interventions [15]. Fusar-Poli et al [2] wrote that “Promotion of good mental health in young people with and without mental disorders has received little empirical research attention and interventions for improving mental health in young people are not well established.” Conducting well-designed effectiveness studies is challenging. However, they are necessary in order to identify interventions effective at improving child and adolescent mental health. Randomized controlled trials are considered the gold standard for evaluating the effectiveness of interventions [18].

After the effectiveness study is conducted, Opp will be made freely available in the Google Play and App Store. Results of the study will continue to be published as they are obtained.

### Limitations

Several limitations will have to be considered when interpreting the results. Both the adolescents and schools are convenience samples, which could lead to selection bias and limit the conclusions that can be drawn from the study [32]. Another limitation is related to the randomization, which will be conducted on a school level and not an individual level [33].

Other limiting factors are related to adherence to the intervention and intervention fidelity. The app does not collect personal data from its users, and it is therefore not possible to objectively track if and how often the adolescents have used the app. However, the intervention group will answer app-related questions on the frequency of use. Both the intervention and control groups receive the same information letter, and participants that are randomized to the intervention group are informed that they can use the app whenever they want and as often as they want, without further instructions. In addition, the intervention group receives a text message with a code to unlock the app after the baseline assessment and randomization.

Another limitation is related to the length of follow-up, which should ideally be longer than 1 month. Kenny et al [15] wrote the following regarding this: “Findings suggest that a 4-week app-based intervention may not be enough to elicit intrapersonal changes in mental health outcomes in a general adolescent population.” Furthermore, the study is based on self-report measures and not on more objective indicators or assessments from other informants, such as parents or teachers, which might be a limitation.

### Conclusion

There are only a few studies that have examined the effects of universal health-promoting mobile apps for adolescents. Therefore, this project will contribute unique knowledge to the field. Opp can theoretically reach up to 420,000 adolescents aged 13 to 19 years in Norway (in 7 cohorts, each with 60,000 adolescents). However, a main challenge will be to implement Opp after its evaluation so that as many adolescents as possible know about the app and can use it if they need to. The findings of this effectiveness study will play an important part in informing decisions on how much effort should be put into implementing Opp in Norway.

## Abbreviations

SDQ: Strengths and Difficulties Questionnaire
WHO-5: World Health Organization–5 Well-being Index

## References

[1] (2001). Strengthening Mental Health Promotion. World Health Organization.

[2] Fusar-Poli P, Salazar de Pablo G, De Micheli A, Nieman DH, Correll CU, Kessing LV, Pfennig A, Bechdolf A, Borgwardt S, Arango C, van Amelsvoort T (2020). What is good mental health? A scoping review. Eur Neuropsychopharmacol.

[3] Barry MM, Barry MM, Clarke AM, Petersen I, Jenkins R (2019). Concepts and Principles of Mental Health Promotion. Implementing Mental Health Promotion.

[4] (1986). Ottawa charter for health promotion. World Health Organization.

[5] Coe G, de Beyer J (2014). The imperative for health promotion in universal health coverage. Glob Health Sci Pract.

[6] Holte A (2012). Ti prinsipper for forebygging av psykiske lidelser [Ten principles for the prevention of mental disorders]. Tidsskr Nor psykolforen.

[7] Guidelines on mental health promotive and preventive interventions for adolescents. World Health Organization.

[8] Blakemore S (2019). Adolescence and mental health. Lancet.

[9] (2020). Children and media. A survey of 9–18-year-olds' digital media habits. Norwegian Media Authority.

[10] Aguirre Velasco A, Cruz ISS, Billings J, Jimenez M, Rowe S (2020). What are the barriers, facilitators and interventions targeting help-seeking behaviours for common mental health problems in adolescents? A systematic review. BMC Psychiatry.

[11] Kenny R, Dooley B, Fitzgerald A (2016). Developing mental health mobile apps: Exploring adolescents' perspectives. Health Informatics J.

[12] Donker T, Petrie K, Proudfoot J, Clarke J, Birch M, Christensen H (2013). Smartphones for smarter delivery of mental health programs: a systematic review. J Med Internet Res.

[13] Karasouli E, Adams A (2014). Assessing the evidence for e-resources for mental health self-management: a systematic literature review. JMIR Ment Health.

[14] Goldberg SB, Lam SU, Simonsson O, Torous J, Sun S (2022). Mobile phone-based interventions for mental health: A systematic meta-review of 14 meta-analyses of randomized controlled trials. PLOS Digit Health.

[15] Kenny R, Fitzgerald A, Segurado R, Dooley B (2020). Is there an app for that? A cluster randomised controlled trial of a mobile app-based mental health intervention. Health Informatics J.

[16] Kuyken W, Ball S, Crane C, Ganguli P, Jones B, Montero-Marin J, Nuthall E, Raja A, Taylor L, Tudor K, Viner RM, Allwood M, Aukland L, Dunning D, Casey T, Dalrymple N, De Wilde K, Farley E, Harper J, Kappelmann N, Kempnich M, Lord L, Medlicott E, Palmer L, Petit A, Philips A, Pryor-Nitsch I, Radley L, Sonley A, Shackleford J, Tickell A, Blakemore S, Team TM, Ukoumunne OC, Greenberg MT, Ford T, Dalgleish T, Byford S, Williams JMG (2022). Effectiveness and cost-effectiveness of universal school-based mindfulness training compared with normal school provision in reducing risk of mental health problems and promoting well-being in adolescence: the MYRIAD cluster randomised controlled trial. Evid Based Ment Health.

[17] Hamdani SU, Zafar SW, Suleman N, Waqas A, Rahman A, Zill-E-Huma, Um-Ul-Baneen (2022). Effectiveness of relaxation techniques 'as an active ingredient of psychological interventions' to reduce distress, anxiety and depression in adolescents: a systematic review and meta-analysis. Int J Ment Health Syst.

[18] Axford N, Berry V, Lloyd J, Wyatt K (2022). How can we optimise learning from trials in child and adolescent mental health?. Evid Based Ment Health.

[19] Kaiser S, Martinussen M, Adolfsen F, Breivik K, Kyrrestad H (2021). An app-based intervention for adolescents exposed to cyberbullying in Norway: protocol for a randomized controlled trial. JMIR Res Protoc.

[20] Høgsdal H, Kaiser S, Kyrrestad H (2023). Adolescents’ assessment of two mental health–promoting mobile apps: results of two user surveys. JMIR Form Res.

[21] (1998). Wellbeing Measures in Primary Health Care/The Depcare Project. World Health Organization.

[22] Goodman R (1997). The Strengths and Difficulties Questionnaire: a research note. J Child Psychol Psychiatry.

[23] Topp CW, Østergaard Søren Dinesen, Søndergaard Susan, Bech P (2015). The WHO-5 Well-Being Index: a systematic review of the literature. Psychother Psychosom.

[24] Hall CL, Guo B, Valentine AZ, Groom MJ, Daley D, Sayal K, Hollis C (2019). The validity of the Strengths and Difficulties Questionnaire (SDQ) for children with ADHD symptoms. PLoS One.

[25] Røysamb E, Schwarzer R, Jerusalem M Norwegian Version of the General Perceived Self-Efficacy Scale 1998. Freie Universität Berlin.

[26] Leganger A, Kraft P, Røysamb E (2000). Perceived self-efficacy in health behaviour research: Conceptualisation, measurement and correlates. Psychol Health.

[27] Tafarodi RW, Swann W B (1995). Self-liking and self-competence as dimensions of global self-esteem: initial validation of a measure. J Pers Assess.

[28] Tafarodi R, Swann W (2001). Two-dimensional self-esteem: theory and measurement. Pers Individ Dif.

[29] Silvera DH, Neilands T, Perry JA (2001). A Norwegian translation of the self-liking and competence scale. Scand J Psychol.

[30] Hysing Mari, Harvey Allison G, Stormark Kjell Morten, Pallesen Ståle, Sivertsen Børge (2018). Precursors of delayed sleep phase in adolescence: a population-based longitudinal study. Sleep.

[31] (2022). Tjenester for Sensitive Data [Services for sensitive data]. University of Oslo.

[32] Henderson M, Page L (2007). Appraising the evidence: what is selection bias?. Evid Based Ment Health.

[33] Esserman Denise, Allore Heather G, Travison Thomas G (2016). The method of randomization for cluster-randomized trials: challenges of including patients with multiple chronic conditions. Int J Stat Med Res.

